# Tissue-resident memory T cells in gastrointestinal tumors: turning immune desert into immune oasis

**DOI:** 10.3389/fimmu.2023.1119383

**Published:** 2023-03-09

**Authors:** Mengjie Liang, Xingzhou Wang, Daming Cai, Wenxian Guan, Xiaofei Shen

**Affiliations:** Department of General Surgery, Affiliated Drum Tower Hospital, Nanjing University Medical School, Nanjing, China

**Keywords:** tissue resident memory T, gastrointestinal tumor, anti-tumor response, cancer immunotherapy, tumor immune microenvironment

## Abstract

Tissue-resident memory T cells (Trm) are a particular type of T cell subgroup, which stably reside in tissues and have been revealed to be the most abundant memory T cell population in various tissues. They can be activated in the local microenvironment by infection or tumor cells and rapidly clean them up to restore homeostasis of local immunity in gastrointestinal tissues. Emerging evidence has shown that tissue-resident memory T cells have great potential to be mucosal guardians against gastrointestinal tumors. Therefore, they are considered potential immune markers for immunotherapy of gastrointestinal tumors and potential extraction objects for cell therapy with essential prospects in clinical translational therapy. This paper systematically reviews the role of tissue-resident memory T cells in gastrointestinal tumors and looks to the future of their prospect in immunotherapy to provide a reference for clinical application.

## 1 Introduction

Immunotherapy has developed rapidly in the past few years and has become one of the first-line treatment strategies in gastrointestinal tumors, of which immune checkpoint inhibitors (ICIs) are the representative drug and have achieved promising therapeutic effects by activating the exhausted immune cells. T cells, especially CD8^+^ T cells, are the main effector cells of the anti-tumor immune response. Memory T cells are the main T cell subsets that exert anti-infection and anti-tumor immune response due to their contact with infection and/or tumor antigens (1). These cells can form long-term memory on tumor-specific antigens, which makes them reliable candidates as a target for anti-tumor therapy. Memory T cells have two subsets: circulating memory T cells and tissue-resident memory T (Trm) cells. In gastrointestinal tissues, Trm cells reside in local tissues, survive for a long time, self-renew, and initiate a rapid response when encountering infection antigens and tumor antigens. At the same time, Trm cells are also considered to be the main population responding to PD-1 inhibitors, playing an important role in immune therapy (2, 3). Therefore, a systematic review of the role of Trm in gastrointestinal tumors will provide an important theoretical basis for better understanding how it plays a positive anti-tumor immune response and optimizes existing immunotherapy strategies.

## 2 Overview of tissue-resident memory T cells

### 2.1 Phenotype of tissue-resident memory T cells

**Figure 1:**
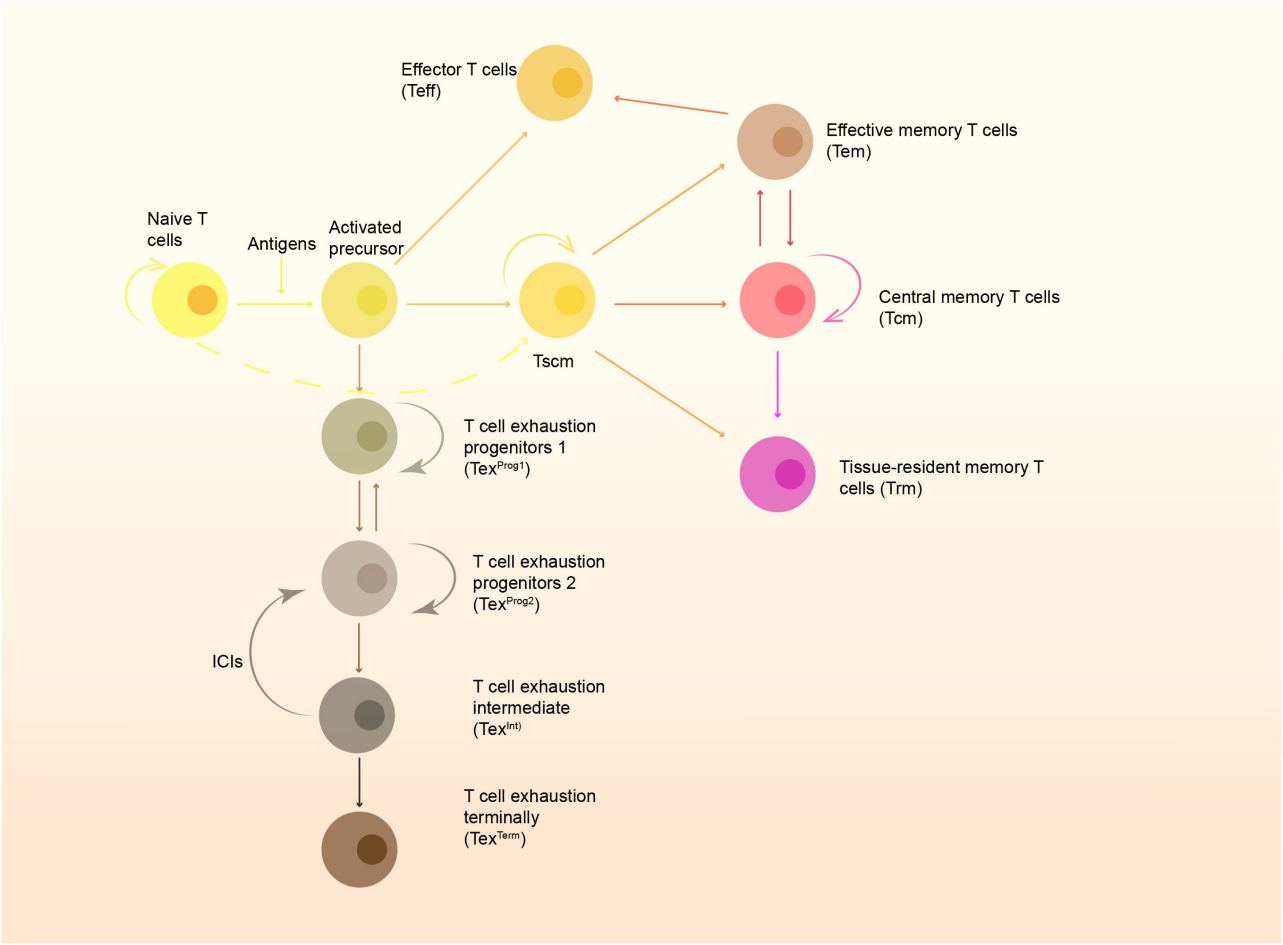
The formation of memory cells and the T cell exhaustion pathway. Naïve T cells are stimulated by antigens presented by APC and develop to active precursors, which further differentiate into effector T cells (Teff) and stem-like memory T cells (Tscm). Tscm can further develop to central memory T cells (Tcm), effector memory T cells (Tem) and tissue-resident memory T cells (Trm). Some of them can also become exhausted gradually, and restoration of function of these cells rely on immune checkpoint inhibitors (ICIs).

Naive T cells can differentiate into memory T cells after being stimulated by antigens. Circulating memory T cells include central memory T cells (Tcm) and effector memory T cells, circulating from circulation, secondary lymphoid organs, and in some situations non-lymphoid tissues (4). Tissue-resident memory T cells were first found in the intestinal mucosa, but were once considered as a special subset of Tem cells (5). Gebhardt et al. later confirmed and officially named this type of memory T cell subset in the skin (6), and now tissue-resident memory T cells have been found in almost all human tissues, including lymph nodes (7). Tissue-resident memory T cells and circulating memory T cells have different phenotypes, molecular characteristics and functions (8) (**Figure 1**): Tcm is relatively static, and mainly exists in lymphatic tissues, highly expressing lymphoid homing receptor. Tcm can be activated when stimulated by antigens, and then proliferate and differentiate into other types of memory T cells (**Figure 1**). Compared to Tcm, Tem mainly exists in non-lymphoid tissues or tissues with inflammation. Mostly, Tcm differentiates into Tem under stimulation. Interestingly, it has been revealed that a small number of Tem cells can differentiate into Tcm cells to maintain stability and its durable effects in the absence of antigen stimulation (1). It used to be thought that Trm cells steadily reside in local tissues to realize rapid immune response to the antigens, with typical characteristics of low homing-related molecule expression and high expression of tissue-resident-associated molecules like CD69 and CD103 (6, 9, 10). Hartana et al. afterwards found a small number of Trm cells in the peripheral blood of healthy people (11), indicating that Trm cells might enter peripheral blood. Some Trm cells can upregulate the expression of molecules such as CD36 to move out of the tissues (12). Beura et al. (13) found that Trm cells in non-lymph node tissues can migrate to lymph nodes, thus increasing the accumulation of antigen-specific Trm cells in lymph nodes, especially in draining lymph nodes. Fonseca et al. (14) also found that the reactivated Trm can re-enter the circulating pool called “ex-Trm”. And these ex-Trm cells retain strong affinity for the original tissue. Fonseca et al. pointed out that Trm cells have certain developmental plasticity and can regenerate Tcm cells and Tem cells, which allows outside-in immune responses. Behr et al. (15) proved this result and they also found that ex-Trm-derived cells showed a higher protective electrical potential than their non-ex-Trm-derived counterparts. Thus, Trm cells retain developmental plasticity and shape both local and systemic T cell responses.

The molecular characteristics of tissue-resident memory T cells generally include high expression of CD103, CD69, and CD49a, as well as low expression of CCR7 and S1PR1, among which the down-regulation of S1PR1 is considered as the precondition for tissue residency of Trm (16). T cells have been found to circulate into the blood in response to sphingosine-1-phosphate (S1P). However, CD69 play an antagoinistic role for S1PR1 (17) by binding to S1P interferes with the transmembrane region of S1PR1 and promoting S1PR1 internalization (18). Joint regulation of CD69 and other important transcriptional pathways (described below) eventually leads to the low expression and activity of S1PR1 in Trm, which can’t respond to S1P. The synergistic effect of low CD62L and high CD69 expression is to prevent the lymphatic homing and migration of Trm cells, which is one of the critical factors for Trm to settle in tissues. Thus tissue-resident memory T cells can also be simply defined as CD62L^-^CD69^+^ T cells to distinguish them from circulating memory T cells. Similarly, low expression CD62L and CCR7 effectively prevent T cells from migrating through the vascular wall (8, 19–21).

**Figure 2:**
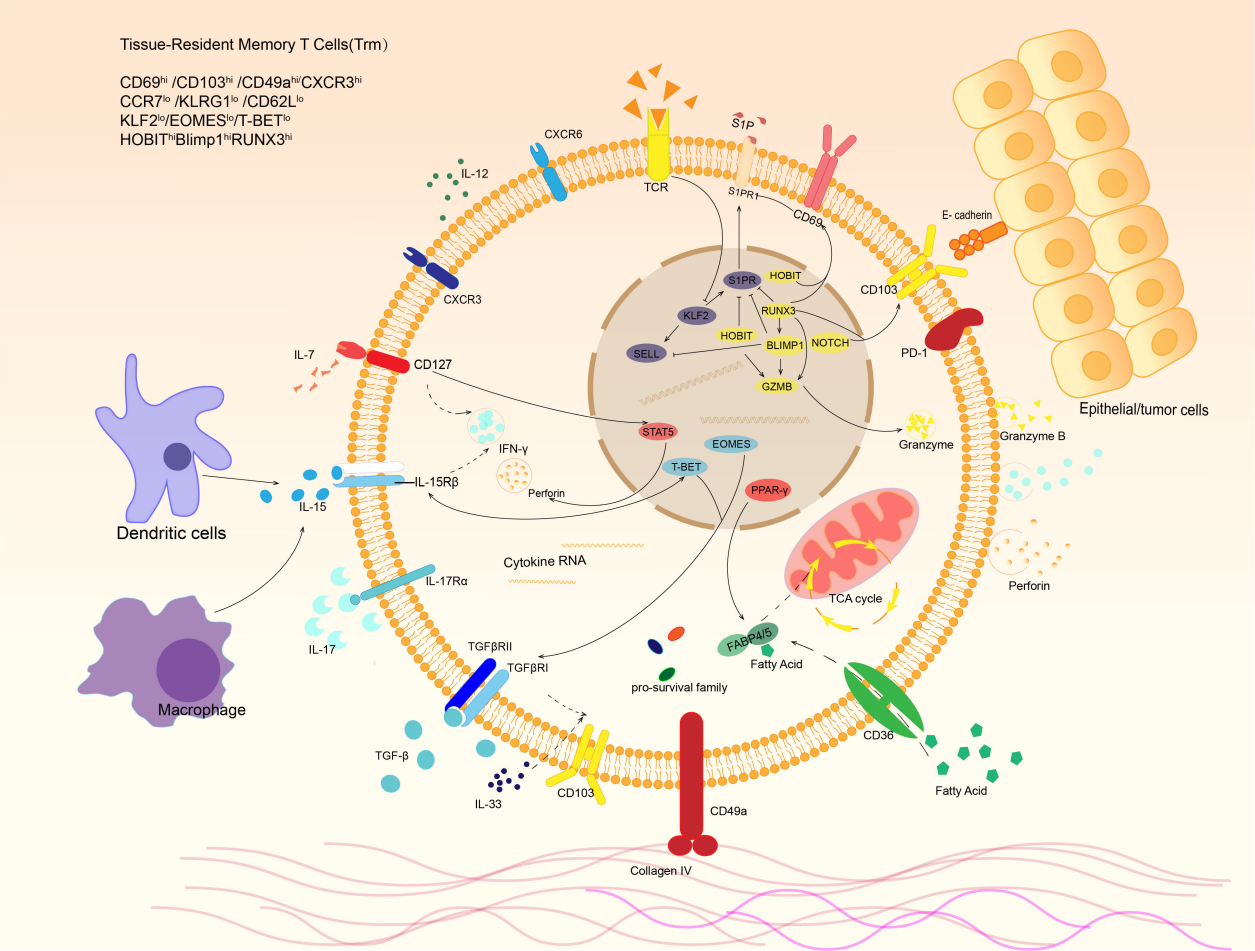
Phenotypes and signaling pathways of tissue-resident memory T cells. Trm cells express distinct phenotype under stimulation of various cytokines in tumor microenvironment. For example, dendritic cells and macrophages can release IL-15, which binds to IL-15 receptor and activates transcription factor T-bet, resulting in IFN-γ expression of Trm. In response to IL-7 in tumor microenvironment, STAT5 is activated and leads to IFN-γand perforin production. E-cadherin expressed by other cells and extracellular matrix like collagen IV are also able to bind the receptors expressed on Trm surface. After that, they release a variety of cytokines to kill targeted cells and regulate local immune microenvironment.

In addition, CD103 and CD49a are considered key tissue residence molecules (20). CD103 can interact with E-cadherin on the surface of epithelial cells to increase the aggregation of Trm in tissues, and induces the response of tissue-resident memory T cells to chemokines (22). Although some tissue-resident memory T cells do not express CD103, molecules with similar effects to CD103 were found in these CD103^-^ Trm cells: in liver, CD103^-^Trm cells can adhere to the hepatic sinusoid epithelium by LFA-1, enabling tissue retention (23). In addition to helping T cells to reside in peripheral tissue, CD69 and CD103 played a significant role in the formation and survival of Trm (24). CD103 may also be related to lipid metabolism by inducing CD36, CD103^hi^Trm often has a higher expression level of CD36 and up-regulation of lipid-related metabolic pathways than CD103^lo^Trm (25). CD49a, the very late antigen-1(VLA-1), also known as integrin α1, can form a complex with CD49d that achieves adhesion to epithelial cells through its ligands, while this adhesion can allow Trm to migrate along the epithelial basement membrane. CD49a promotes the survival of Trm and is involved in the regulation of IL-15-induced production of granzyme and perforin to maintain the functional stability of Trm (20, 26, 27). Cytokines in the immune microenvironment, such as IL-2/IL-12/IL-15/IL-7, regulate CD62L and S1PR1 and play an essential role in the formation, survival, function maintenance, and regeneration of Trm in tissues (**Figure 2**).

With the development of sequencing technology, the heterogeneity of Trm has been revealed, which helps us better understand how Trm cells play the role in local tissues. As mentioned above, CD103 is essential for Trm residence and maintenance. CD103^+^ Trm cells are the main force in gastrointestinal tissues to fight against infection and tumors, while CD103^-^ Trm cells have a more unique function. For instance, in the intestine, CD103^+^CD69^+^CD8^+^ Trm cells possess high ability to produce inflammatory cytokines, while CD103^-^CD69^+^CD8^+^ Trm cells have a high expression of β2-integrin and are more able to produce granzyme (28). Besides, researchers have found that CD103^-^ Trm can differentiate into CD103^+^ Trm to mediate immune response towards secondary infections, instead of the primarily resident CD103^+^ Trm (29, 30). Besides the different function, various subsets of Trm cells, including CD103^+^CD69^+^, CD103^-^CD69^+^ and CD103^-^CD69^-^ Trm cells, have shown great potential to proliferate. Further exploration

Transcription factors involved in Trm formation include high expression of Runx3, Hobit, Blimp1 and Notch, with low expression of T-bet, Eomes and Klf2. In addition, Trm also expresses pro-survival family members such as Bcl-2 and various anti-apoptotic factors (19, 21, 31). The expression of Runx3 is considered a key factor for the formation of Trm. Overexpression of Runx3 during thymic T cell development promotes the formation of CD8^+^T cells and is also an important factor in the induction of CD103 (32). And effector T cells in tissues tend to have higher Runx3 expression (33). Runx3 can promote the expression of tissue-residency genes and molecules (CD69, Blimp-1, etc.) and suppress genes related to tissue egress and recirculation (T-bet, S1PR1, Klf2, etc.) (34, 35). Blimp-1, one of the downstream targets of Runx3, can also regulate the expression of S1PR1 and CD62L directly or indirectly through Klf2 and Sell, while Hobit, a homolog of Blimp-1, has a similar function (20, 21, 31, 36). Notch can also promote the expression of CD103 (37). In contrast, TCF-1, a transcription factor maintaining CD8^+^ T cell stemness, can repress CD103 expression by directly suppressing the transcription and inhibiting TGF-β-induced CD103 expression, which restricts Trm development (38). Hobit may maintain Trm production in the gut and skin in the absence of Blimp-1 (36). The downstream targets of Notch are solute carriers of nutrients, including amino acids, indicating that Notch helps maintain the nutritional metabolism of Trm (39). In addition to regulating the formation of tissue-resident phenotypes, these transcription factors also control the cytotoxicity of Trm. Runx3 promotes the differentiation of cytotoxic T lymphocytes (CTLs) and inhibits terminal differentiation, ensuring the development, growth, and longevity of CTLs (34). At the same time, Runx3, together with Blimp-1, Hobit, T-bet and others, regulates the production of granzyme B and/or perforin through its complex regulatory pathway (40). It is worth noting that Hobit is superior to Blimp-1 in long-term expression of granzyme (41). Eomes was thought to suppress Trm cell formation, but a recent study revealed that Eomes was essential for Trm maintenance in the intestine but not the colon (42). Thus, various expression of transcriptional factors determines heterogeneity and existence of Trm cells, which can be altered in case of tumor or infection. Focusing on the alteration may help us better to understand the phenotype and function changes of Trm cells and provide more ideas to regulate these cells to play an anti-tumor role.

### 2.2 Anti-tumor effect of tissue-resident memory T cells and T cell exhaustion

Trm cells have been confirmed to monitor of local immunity and play an important role in infectious diseases (6, 19, 43–45) and autoimmune diseases (46, 47). The significant negative correlation between *ITGAE* (encoding CD103) and *CD69* gene expression and molecular markers of glioblastoma (48) suggests that CD8^+^Trm cells may have an anti-tumor effect, as demonstrated by studies in other tumors (9, 44, 49, 50). At the same time, tissue-resident memory T cells are also a key target for the regulation of immune-checkpoint blockade (ICB) therapy (22, 51, 52). The anti-tumor effect of Trm is manifested in two aspects: 1. Trm mediates local immunity, and 2. It kills tumor cells by toxicity (53). Activated Trm can identify the tumor-associated antigens and secrete cytotoxic granular proteins, CCL3, CCL4, CCL5 and other chemokines, as well as pro-inflammatory cytokines such as interferon-γ (IFN-γ) and tumor necrosis factors (TNF) (53, 54). The anti-tumor effect of tissue-resident memory T cells is closely related to their phenotypic molecules (**Table 1**). The interaction between CD103 and cadherin protects the survival of Trm in tumor tissues, and also causes the bidirectional signal of T cell receptor (TCR) [24], and induces TCR-dependent tumor antigen-specific killing effect. The release of TNF-γ can promote the up-regulation of vascular adhesion molecule 1(VCAM-1), recruit CD8^+^ circulating memory T cells and B cells, and further induce and promote local immune response (22, 43). In the mouse melanoma model, the defect or inhibition of CD103 manifests as the immune system’s imbalance in regulating tumor growth. Compared with the CD103 wild type mouse, the melanoma in the CD103 deficient mouse is large and grows faster (67). In addition, the cells with high CD103 expression are often accompanied by the expression of CD69 and PD1. CD103^+^Trm also has higher cytotoxicity than CD103^-^Trm (57, 58, 61). CD49a has a similar effect to CD103. After using CD49a inhibitors, the mice in the experimental group also showed uncontrolled tumor growth (56).

**Table 1:** Overview of Trm phenotypes and functions in various tumors.

Tumor	Phenotype	Inhibitory receptors	Value of Trm	Reference
Breast cancer	CD103^+^	PD-1 CTLA-4	Trm genotype was correlated with better prognosis and up-regulated after PD-1 blockade	(9)
Melanoma	CD103^+^CD69^+^	PD-1 CTLA-4 2B4	The increase in the number of tumor-resident memory was related to an improved survival with immunotherapy	(55)
	CD45a/VLA-1^+^	Undetected	Associated with better survival	(56)
Gastric cancer	CD103^+^	PD-1	4-1BB co-stimulation can enhance the Trm regeneration mediated by PD-1 blockade; Low level of tumor infiltration Trm is correlated with low survival rate.	(57)
	CD103^+^	PD-1	CD103^+^CD8^+^T cells have a better prognostic value; High infiltration was associated with better adjuvant chemotherapy.	(58)
		Undetected		(59)
Glioblastoma	CD103^+^CD69^+^	Undetected	prolonged survival	(48)
Esophageal squamous cell carcinoma	CD103^+^	PD-1 TIM-3 TIGIT LAG-3	Positively correlated with the overall survival rate; Induced to effectively inhibit tumor after PD-1 blockade	(3)
Ovarian cancer	CD103^+^	Undetected	Associated with a better prognosis.	(60)
Vaginal melanoma	CD103^+^CD8^+^	PD1 CTLA4	The highest tumor response potential, and proliferation with ICB therapy; Associated with prolonged survival	(2)
Lung cancer	CD103^+^	PD-1 TIM-3 LAG-3 CTLA-4	Predictive of a better survival outcome	(61)
Head and neck cancer	CD49a^+^CD103^+^	PD-1 Tim-3	better overall survival; biomarker for the efficacy of cancer vaccine.	(62)
Hepatocellular carcinoma	CD69^+^CD103^+^CD57^+^	PD-1 TIM-3 CD39	More effective anti-tumor effect and related to the relapse-free survival.	(63)
	CD103^+^Trm, CD8^+^Tex	PD-1 CTLA-4 LAG-3	Trm/Tex was associated with survival	(64)
Colorectal cancer	CD39^+^CD103^+^	PD-1 Tim-3	Predicting the response to ICB therapy and prognosis	(65)
		PD-1 CTLA4		(66)
**urinary bladder cancer**	CD103^+^CD69^+^	PD-1	potential new targets for cancer immunotherapy	(11)

The function of tissue-resident cells is regulated by other immune cells. Dendritic cells promote the differentiation and formation of tissue-resident memory T cells (68) and play a protective role in tumors. The interaction between CXCR6^+^Trm and CXCL16^+^APC is important for maintaining tumor immunity (69). However, some immune cells play a negative regulatory role, tumor-associated fibroblasts (CAFs) are essential in tumor microenvironment and have complex relationships with tumor cells and TILs. CAFs can inhibit Trm activity by upregulating the expression of PD-1 by cytokines, and increase the risk of lymphatic metastasis (70). In addition, Treg cells can inhibit Trm cell immune activity and promote tumor growth, and they have more significant infiltration in the tumor, especially tumor matrix (71, 72). These two immune cell subsets can work together with macrophage type 2 to form an immune barrier against anti-tumor immune response by Trm (72).

Trm has an excellent anti-tumor effect, but like other T cell subsets, it will gradually lose its immune activity and convert into exhausted T cells (Tex) when continuously stimulated by antigens and/or inflammatory factors. According to the expression of Ly108 and CD69, T cells are divided into four stages (**Figure 1**). The intermediate Tex subset (Tex^Int^) will gradually lose its cytotoxicity and proliferation capacity. However, T cells at this stage can regain their cytotoxic effect after PD-1 blockade. Once the cells enter the terminally exhausted subset, T cells will no longer respond to immune checkpoint inhibitors (ICIs) (73). It is now generally accepted that exhausted T cells are characterized by low antigen-stimulated proliferation, high expression of multiple immune checkpoints and progressive loss of T-cell effector function. Thus, immune checkpoints are also known as exhausted-related molecules (74). Trm cells also express multiple immune checkpoints, such as PD-1, TIM-3, and CTLA-1, etc. Interestingly, although Trm cells have multiple immune checkpoint molecules, many studies have shown that their function is not exhausted (11, 75). On the contrary, the functional Trm is more powerful and metabolically active than Tex (64). But it is shown in multiple tumor models that Trm is a group of cells with the trend of exhaustion (9, 11, 53, 64, 75). Zheng et al. (76) performed single-cell sequencing on a variety of tumor-infiltrating T cells, including gastric cancer and esophageal cancer, and found that the exhausted T cells were mainly from effector memory T cells and tissue-resident T cells. Recent study has found that the exhaustion of CD8^+^ T cells is accompanied by an increase in the number of Tc17 cells with poor cytolytic capacity (77).The scRNA-seq of gastric tumor also showed Tc17 exhaustion pathway originating from Trm (78). Another single-cell sequencing results showed that Trm cells could be divided into three subsets: Hobit-enriched CD103^hi^PD-1^lo^Trm, Granzyme K-enriched CD103^lo^PD-1^hi^ Trm, and CD103^hi^ PD-1^hi^CTLA^hi^LAG3^hi^TIGIT^hi^ Trm, while the last subset is considered as exhaustion-related subtype (79). Milne et al. also identified subsets of memory T cells with resident gene expression characteristic of progenitor exhaustion T (Tpex) cell (Id3^hi^Blimp1^lo^) and terminal exhaustion (80), and this study indicates that there exists a complete developmental pathway to exhaustion in Trm. In general, Trm cells may keep their function with the expression of immune checkpoints, but they still can convert into exhausted T cells. The differentiation pathways and intrinsic mechanism remain unclear, and the exploration to the pending issue may provide novel strategies for anti-tumor therapy.

Dysfunction of T cells in the advanced tumor microenvironment is related to tumor immune escape. Once it happens, the number of T cells expressing either high-level or low-level cytotoxic granulosa proteins is significantly reduced and the exhausted-related molecules are significantly overexpressed in tumor microenvironment (TME) (57). Studies have pointed out that non-terminally Trm-Tex dynamic is related to tumor prognosis (63, 64, 76). And the population with high expression of cell cycle-related genes, though less, is likely to be related to the tumor-associated antigen (TAA) (81), and replenish effector Trm cells as they become exhausted (81, 82)

## 3 Tissue-resident memory T cells in gastrointestinal tumors

### 3.1 Tissue-resident memory T cells in gastric cancer

In normal gastric tissue, lamina propria mononuclear cells (LPMCs) have a tissue-resident phenotype, with more than 70% of LPMC CD8^+^ T cells co-expressing CD103 and CD69, and nearly 20% expressing CD103 alone (83). CD69^+^CD103^+^ Trm also accounts for approximately 30% of TILs in the TME of gastric adenocarcinoma with high expression of PD-1, TIGIT, and CD39 (49). Like most solid tumors, Trm cells in gastric cancer have significant spatial heterogeneity. In gastric cancer tissues, more GZMB^-^ Trm cells can be seen, and CD103^-^ T cells infiltrate more in the tumor matrix, while CD103^+^ Trm cells appear more excessively in the tumor epithelium. Various types of Trm are decreased in the tumor matrix with lower activity and function (51). Besides, the infiltration of CD103^+^CD8^+^ Trm cells in advanced gastric cancer is also less than in early gastric cancer (57).

It has been found that development of gastric cancer is associated with dysfunction or declined abundance of Trm (84–86). Trm cells in the tumor microenvironment ingest free fatty acid (FFA) as the main energy source mainly through CD36-FABP4/FABP5 related metabolic pathways (25). However, gastric cancer cells also have up-regulated CD36 and lipid metabolism (87). Aoki (88) and Pan (87) found high expression of CD36 may be associated with peritoneal metastasis of gastric cancer. Furthermore, this suggested that there might be a competitive relationship on lipid uptake between gastric cancer cells and Trm. Recently, Lin et al. (49) revealed that gastric cancer cells deprive Trm of fatty acids through more aggressive competitive uptake, which leads to Trm death. They further found that blocking PD-1/PD-L1 reduced lipid metabolism of gastric cancer cells, but enhanced the lipid uptake and lipid metabolism of Trm cells.

In recent years, tertiary lymphoid structure (TLS) has been found to be associated with the prognosis or development of tumors. TLS is associated with survival and clinical outcomes in patients with immunotherapy for tumors and can serve as a potential therapeutic target for tumors (89, 90). And TLS is also associated with a better prognosis for gastric cancer. Interestingly, Mori et al. found that CD103^+^CD8^+^Trm cells were found in and around TLS, which could secrete high levels of cytolytic enzyme and IFN-γ, and IFN-γ was considered to have the function of promoting TLS formation. Consistently, patients with CD103 overexpression also tend to have more abundant TLS. Patients with CD103^hi^TLS^hi^ also had a better therapeutic response to nivolumab (86, 91).

In addition to the susceptibility and immune microenvironment changes caused by genetic susceptibility, pathogenic microbial infection, and microbiome alterations also play an important role in the occurrence and development of gastric cancer and affect the function of TILs. Gastric cancer is one of the few tumors related to infection, *Helicobacter pylori* (*H. pylori.*) and Epstein–Barr virus (EBV) are the main infection-related factor (92–94). *H. pylori* can inhibit the CD8^+^ CTLs by myeloid-derived suppressor cells (MDSCs) and induce the up-regulated expression of PD-L1 of tumor epithelial cells and macrophages, thereby inducing the resistance of gastric cancer to ICB therapy against PD-1/PD-L1 (95, 96). Xu et al. have shown that the induction of Trm proliferation and differentiation can enhance the local immune response to *H. pylori* (97). A recent study has shown that CagA-specific CD8^+^ Trm cells can infiltrate the gastric mucosa and control the *H. pylori.* infection (98). EBV infection can induce DNA demethylation of host cells, which results in expression of tumor suppressor genes, loss of tumor-related antigens, and over-expression of immune checkpoint like PD-L1 and PD-L2 and induces immune evade of Epstein–Barr virus-associated gastric cancer (EBVaGC) by PD-1/PD-L1 interactions between tumor cells and T cells (99, 100). The typical characteristics of the gastric cancer microbiota were the flora change and the decrease in microbial diversity. The mouse model showed that enhanced *Methylobacterium* in gastric cancer promoted tumor progression by reducing the infiltration of TGF^-^β^+^CD8^+^ Trm and was related to the exhaustion of Trm (101). Similarly, Yang et al. (102) showed higher CXCL13 production in CD8^+^ Trm, which contributed to TLS formation.

### 3.2 Tissue-resident memory T cells in colorectal cancer

In normal intestinal tissue, the proportion of CD69^+^CD103^+^CD8^+^ T cells in CD8^+^ T cells can even exceed 80% (28). However, they have significant functional differences in the lamina propria and epithelial layer. The tissue-resident memory T cells have active pathways that regulate cell survival and cytokine signal transduction in the lamina propria with higher expression of Runx3,NR4A2,ICOS and LITAF, while they show more obvious cytotoxic characteristics in the epithelial layer with a higher level of GZMM,LTB,GZMA and CXCR3 (103). These CD69^+^CD103^+^ Trm cells have been found to highly express CD161 and CD127 (IL-7R), which help maintain T-cell cytotoxicity. In addition, IL-7 can restore the activity of CD8^+^ T cells by reducing the expression of PD-1 (104).

The immune microenvironment regulates the function of CD8^+^ T cells and the formation of CD8+ Trm: TGF-β can inhibit the migration of CD8 T cells, which, together with IL-33, can promote T cell differentiation into Trm (105), TGF-β can induce the synthesis of CD103 and CD69 molecules in the maintenance phase of Trm cells (22, 106). Once TGF-β is blocked, the abundance of Trm cells can significantly decrease (62). Paradoxically, TGF-β has two sides to tumor immunity, and it can regulate the expression of tumor cell cycle-related proteins and inhibit cell cycle from exerting anti-tumor effects in the early stage of cancer. It becomes a spy for immune responses to advanced tumors. When the tumor enters the advanced stage, the tumor cells can secrete a large amount of TGF-β to damage T cell mitochondrial respiration and inhibit the production of IFN-γ, thereby reducing T cell activity and helping the tumor escape through the Treg cell pathway (107, 108). IL-7 appeared to inhibit the negative effects of TGF-β on suppressing Trm development (104). Intraepithelial lymphocytes (IELs) are activated by IL-15 (109), and IL-15 can mediate their proliferation and survival, and regulate the expression of IFN-γ (110, 111). IL-15 can also regulate the growth and apoptosis of colorectal cancer tumor cells, and low level of IL-15 is also associated with low-infiltrating lymphocytes in tumor microenvironment and poor prognosis of the colorectal cancer (112). Desboi et al. (113) found that IL-15 hyper-agonist receptor-linker -IL-15 (RLI) could limit tumor growth in the early stage of colorectal cancer and the combined use of PD-1 inhibitors had a higher activation rate of CD8^+^ TILs in colorectal cancer compared with PD-1 inhibitors or RLI alone, but had no significant inhibition or CD8^+^ TILs activation for advanced colorectal cancer, which was consistent with T cell exhaustion.

The prognosis of colorectal cancer (CRC) is also related to its subtypes. Microsatellite instability (MSI) is considered as one of the main carcinogenic pathways of colorectal cancer and can be used as a prognostic marker. According to the mismatch repair (MMR) and MSI, RCRs can be divided into high microsatellite instability RCRs (MSI-H)/mismatch repair system defective CRC (dMMR), low microsatellite instability RCRs (MSI-L) and microsatellite stability RCRs (MSS). Colorectal patients with MSS/MSS-L have a worse prognosis and respond to targeted PD-1/PD-L1 or CTLA-4, while the sensitivity to ICIs and prognosis of those with dMMR/MSI-H are significantly better (114–116). Compared with normal intestinal mucosa or MSS-RCR tissue, CD8^+^ T cells have more significant enrichment in MMR-deficient CRC tissue (117, 118). Studies have found that CD8^+^ Trm in colorectal cancer patients has specific demethylation of CD103 and CD39-related genes (65), CD103^+^CD39^+^ T cells have a strong MHC-dependent tumor killing ability, and the co-expression of CD103 and CD39 is also considered to be the key to T cell tumor-specific recognition characteristics and differentiation from bystander T cells, and can be used as an independent risk factor for the prognosis of colorectal cancer (65, 119).

CRC is associated with environmental factors and earlier onset age has been observed among these patients (120, 121). In most cases, the adenoma-carcinoma-metastasis process is experienced, which is associated with the accumulation of specific genetic events of “APC-KRAS-TP53”. Particular case was reported that the lung metastases in a patient with metastatic colorectal cancer disappeared or shrank after autologous isolation and culture of CD8^+^ T cells that had a specific effect on the mutant KRAS gene, and were still in a clinically disease-free state four months after surgery (122), which indicated the effectiveness and broad prospect of Trm as an as a potential target of colorectal cancer adoptive cell therapy.

## 4 Application of tissue-resident memory T cells in gastrointestinal tumors

### 4.1 Prognostic value and ICIs response of tissue-resident memory T cells

Tissue-resident memory T cells are associated with the prognosis of a variety of tumors (**Table 1**). In general, a high proportion of CD8^+^ Trm predicts better survival, and has better prognostic value than the total number of CD8^+^ T cells. Further studies show that non-terminal tissue-resident memory T cells may be more important (63); and it has also been found in ovarian cancer that its immunogenicity is determined by fewer stem cell-like tissue-resident memory T cells (82). Tissue-resident memory T cells, perhaps the highest tumor reaction potential T cells, are important participants in the anti-tumor effects, and can respond to ICIs in the early stage (123, 124). PD-1 blockade can significantly proliferate tissue-resident memory T cells with an enhancement of anti-tumor effect (2), and it also promotes the migration of Tcm into the tumor microenvironment and differentiation into Trm (125). Single-cell sequencing analysis also showed significant up-regulation of tissue presence-related phenotypes after blocking PD-1 (9). Tumor microenvironment in patients with better ICIs responses tends to be more Trm-rich (55), and it has been proved that highly infiltrated tissue-resident memory T cells can be used as a marker of benign ICB therapeutic response in colorectal cancer (65, 66), breast cancer (9, 50), melanoma (55) and other tumors (60). In addition, tissue-resident memory T cells can be highly enriched in the tumor microenvironment after chemoradiotherapy (126) and are associated with a good prognosis for patients who receive doublet chemotherapy after surgery (59), suggesting that ICIs combined with adjuvant chemoradiotherapy can achieve better anti-tumor effects. In fact, Phase III clinical trials have shown that Nivolumab combined with neoadjuvant chemotherapy significantly improved the overall survival and progression-free survival of patients with gastric cancer (127).

### 4.2 Enhancing activity and T cell therapy of tissue-resident memory T cells

Given the excellent anti-tumor effect and potential prognostic value, it is worth exploring how to make better use of tissue-resident memory T cells for anti-tumor therapy. And it also implies a better anti-tumor environment in the gastric cancer microenvironment because of a lower proportion of terminal-exhausted CD8^+^ T cells and a higher proportion of CD8^+^ Trm in the gastric cancer microenvironment (76). Enhancing the proliferative capacity and cytotoxicity of Trm may be a better option for gastrointestinal tumor immunotherapy by the combination of 4-1BB co-stimulation or RLI with PD-1 blockade at this stage (57, 113).

CD8^+^ T cells can be induced to express CD103 by reprogramming tumor-infiltrating DCs with β-glucan gel polysaccharide. CD103^+^CD8^+^ T cells generated in this way could control the growth of existing tumors (128). In addition, it has been reported that a patient with locally recurrent gastric cancer was relieved by injecting DCs into tumor for 30 months (129). HBV-specific tumor-resident T cells correlated with relaxation-free survival of hepatocellular carcinoma (63), considering the relationship between gastric cancer and infection factors, as well as the particularity of gastrointestinal flora, the induction of infection-derived specific tissue-resident memory T cells by vaccine may have exploratory significance for preventing the occurrence of gastrointestinal tumors or intervening in their development (130). Induction the formation of Trm *via* cancer vaccines is also conceivable (62, 131, 132)

Autologous tumor infiltrating lymphocytes adoptive therapy has observed objective regression or even complete remission of tumors in melanoma (133) and breast cancer (134) with objective tumor regression, or even complete remission. As an important subset of TILs, Trm may be able to undertake this role better. Anada and Matthewd’s research showed that the expression of *Runx-3* promoted the formation of tissue-resident memory T cells, and they use melanin model revealed that the metastasis and localization of CD8^+^ T with high expression of *Runx-3* in TME inhibited the growth of tumor (35). As mentioned above, a part of recyclable tissue-resident memory T cells does exist in the human body (14, 15), which indicate that constructing T cells by inducing or coercing the expression of genes related to tissue retention may be one of the feasible pathways for cell therapy.

Although adoptive T cell therapy has made great achievements and shown great potential, how to select more tumor antigen-specific T (Tas) cells or induce the expansion of Tas cells is still a challenge. The lack of tumor antigen specificity of CAR-T increases the potential risk of targeted tumor removal toxicity, and even serious side effects (135). As mentioned above, tissue-resident memory T cells in the gastrointestinal tumor microenvironment have a significant portion co-express CD39 and CD103 (49, 65), which is considered to be a marker of tumor antigen-specific response in solid tumors (119). In addition, the TCR of Tas cells in patients is a more personalized choice of TCR-T cell therapy. A transcriptomic profiles of neoantigen-reactive T cells from gastrointestinal tumors also revealed that most neoantigen-reactive cells co-express CXCL13 and GZMA (136). He et al. proved that CXCL13 is a unique marker of Tas cells and they found ENTPD1 (CD39) can be used as a surface marker of CD8^+^ Tas cells. Moreover, TCR-T cells expressing TCR from Tas cells show significant therapeutic effects on autologous patient-derived xenograft (PDX) tumors from autologous patients (137). Therefore, rational utilization of the tissue-resident property of CXCL13^+^ Trm may lead to more precise targeted tumor therapy. Since it is relatively difficult to extract Trm in local tissues, Trm in tumor draining lymph nodes would also be a good alternative. Trm cells have been proved to exist in not only non-lymphoid tissues but also lymphoid tissues, especially lymph node (7), and infection-associated antigen-specific CD8^+^ Trm within the lymph nodes can be migrated from non-lymph node tissue (13). Recent studies also showed that tumor-specific CD8^+^ Trm existed in draining lymph nodes and could prevent the spread of melanoma in the lymph nodes (138). Hence, the tumor-specific Trm located in lymph node can not only prevent tumor cells spreading, but also can be an alternative option for extraction and TCR repertoire recognition and following TCR-T transformation.

## 5 Concluding remarks

Immunotherapy for gastrointestinal tumors remains a worldwide challenge. Immunotherapy for cancer aims to identify and eliminate tumor cells by stimulating T cells with tumor-killing effects to reach the tumor microenvironment. Tumor-infiltrating tissue-resident T cells can be used as a potential target population of ICIs, and it is also a biomarker for the prognosis of gastrointestinal tumors and PD-1 blocking effect. The phenotype and function of tissue-resident memory T cells implicate their potential therapeutic value. First, Trm resides in peripheral non-lymphoid tissues, and the reoccurring immune response is much faster than that of circulating memory T cells, thus enabling Trm to participate in immune regulation of tumor more quickly. Second, CD8^+^ Trm can secrete a variety of cytokines to promote tumor cell lysis and death, and the related phenotypic molecules can also exercise a regulatory effect on tumor growth. Therefore, we judged that the tissue-resident memory T cells could be potential extraction targets for adoptive T cell therapy. However, as we have mentioned repeatedly above, T cell exhaustion is closely related to the function of Trm and immune escape of tumor and Tex^Term^ has lost its immune activity. Besides, tumor tissues also contain T cell populations without tumor immune function such as bystander T cells. Therefore, in order to better utilize Trm for anti-tumor, we need more details how Trm cells’ functions are restricted by tumor and find out the cell populations with tumor antigen specificity and long-term stable cytotoxic effect among numerous subsets or produce effective targeted anti-tumor T cells by cell technology to intervene in the exhaustion of CD8^+^ Trm by certain means and implement more accurate regulation for this crimp.

## Author contributions

ML, XW: Writing - original draft. DC: Figure drawing. XS and WG: Conceptualization, Writing – review & editing, funding acquisition. All authors contributed to the article and approved the submitted version.

## References

[1] Muroyama Y Wherry EJ. Memory T-cell heterogeneity and terminology. Cold Spring Harb Perspect Biol (2021) 13(10):a037929. doi: 10.1101/cshperspect.a037929 33782027 PMC8485749

[2] Pizzolla A Keam SP Vergara IA Caramia F Thio N Wang M. Tissue-resident memory T cells from a metastatic vaginal melanoma patient are tumor-responsive T cells and increase after anti-PD-1 treatment. J Immunother Cancer (2022) 10(5):e004574. doi: 10.1136/jitc-2022-004574 35550554 PMC9109124

[3] Han L Gao Q-L Zhou X-M Shi C Chen G-Y Song Y-P. Characterization of CD103 CD8 tissue-resident T cells in esophageal squamous cell carcinoma: May be tumor reactive and resurrected by anti-PD-1 blockade. Cancer Immunol Immunother CII (2020) 69:1493–504. doi: 10.1007/s00262-020-02562-3 PMC11027643 32285170

[4] Sallusto F Geginat J Lanzavecchia A. Central memory and effector memory T cell subsets: function, generation, and maintenance. Annu Rev Immunol (2004) 22:745–63. doi: 10.1146/annurev.immunol.22.012703.104702 15032595

[5] Masopust D Vezys V Marzo AL Lefrançois L. Preferential localization of effector memory cells in nonlymphoid tissue. Sci (New York NY) (2001) 291:2413–7. doi: 10.1126/science.1058867 11264538

[6] Gebhardt T Wakim LM Eidsmo L Reading PC Heath WR Carbone FR. Memory T cells in nonlymphoid tissue that provide enhanced local immunity during infection with herpes simplex virus. Nat Immunol (2009) 10:524–30. doi: 10.1038/ni.1718 19305395

[7] Schenkel JM Fraser KA Masopust D. Cutting edge: resident memory CD8 T cells occupy frontline niches in secondary lymphoid organs. J Immunol (2014) 192:2961–4. doi: 10.4049/jimmunol.1400003 PMC3965619 24600038

[8] Martin MD Badovinac VP. Defining memory CD8 T cell. Front Immunol (2018) 9:2692. doi: 10.3389/fimmu.2018.02692 30515169 PMC6255921

[9] Savas P Virassamy B Ye C Salim A Mintoff CP Caramia F. Single-cell profiling of breast cancer T cells reveals a tissue-resident memory subset associated with improved prognosis. Nat Med (2018) 24:986–93. doi: 10.1038/s41591-018-0078-7 29942092

[10] Yang K Kallies A. Tissue-specific differentiation of CD8(+) resident memory T cells. Trends Immunol (2021) 42:876–90. doi: 10.1016/j.it.2021.08.002 34531111

[11] Hartana CA Ahlén Bergman E Broomé A Berglund S Johansson M Alamdari F. Tissue-resident memory T cells are epigenetically cytotoxic with signs of exhaustion in human urinary bladder cancer. Clin Exp Immunol (2018) 194:39–53. doi: 10.1111/cei.13183 30009527 PMC6156818

[12] Klicznik MM Morawski PA Höllbacher B Varkhande SR Motley SJ Kuri-Cervantes L. Human CD4(+)CD103(+) cutaneous resident memory T cells are found in the circulation of healthy individuals. Sci Immunol (2019) 4(37):eaav8995. doi: 10.1126/sciimmunol.aav8995 31278120 PMC7057121

[13] Beura LK Wijeyesinghe S Thompson EA Macchietto MG Rosato PC Pierson MJ. T Cells in nonlymphoid tissues give rise to lymph-Node-Resident memory T cells. Immunity (2018) 48:327–38.e5. doi: 10.1016/j.immuni.2018.01.015 29466758 PMC5828517

[14] Fonseca R Beura LK Quarnstrom CF Ghoneim HE Fan Y Zebley CC. Developmental plasticity allows outside-in immune responses by resident memory T cells. Nat Immunol (2020) 21:412–21. doi: 10.1038/s41590-020-0607-7 PMC7096285 32066954

[15] Behr FM Parga-Vidal L Kragten NAM van Dam TJP Wesselink TH Sheridan BS. Tissue-resident memory CD8+ T cells shape local and systemic secondary T cell responses. Nat Immunol (2020) 21:1070–81. doi: 10.1038/s41590-020-0723-4 32661361

[16] Skon CN Lee JY Anderson KG Masopust D Hogquist KA Jameson SC. Transcriptional downregulation of S1pr1 is required for the establishment of resident memory CD8+ T cells. Nat Immunol (2013) 14:1285–93. doi: 10.1038/ni.2745 PMC3844557 24162775

[17] Shiow LR Rosen DB Brdicková N Xu Y An J Lanier LL. CD69 acts downstream of interferon-alpha/beta to inhibit S1P1 and lymphocyte egress from lymphoid organs. Nature (2006) 440:540–4. doi: 10.1038/nature04606 16525420

[18] Baeyens A Bracero S Chaluvadi VS Khodadadi-Jamayran A Cammer M Schwab SR. Monocyte-derived S1P in the lymph node regulates immune responses. Nature (2021) 592:290–5. doi: 10.1038/s41586-021-03227-6 PMC8475585 33658712

[19] Schenkel JM Masopust D. Tissue-resident memory T cells. Immunity (2014) 41:886–97. doi: 10.1016/j.immuni.2014.12.007 PMC4276131 25526304

[20] Crowl JT Heeg M Ferry A Milner JJ Omilusik KD Toma C. Tissue-resident memory CD8(+) T cells possess unique transcriptional, epigenetic and functional adaptations to different tissue environments. Nat Immunol (2022) 23:1121–31. doi: 10.1038/s41590-022-01229-8 PMC10041538 35761084

[21] Dijkgraaf FE Kok L Schumacher TNM. Formation of tissue-resident CD8(+) T-cell memory. Cold Spring Harb Perspect Biol (2021) 13(8):a038117. doi: 10.1101/cshperspect.a038117 33685935 PMC8327830

[22] Corgnac S Boutet M Kfoury M Naltet C Mami-Chouaib F. The emerging role of CD8(+) tissue resident memory T (TRM) cells in antitumor immunity: A unique functional contribution of the CD103 integrin. Front Immunol (2018) 9:1904. doi: 10.3389/fimmu.2018.01904 30158938 PMC6104123

[23] Franciszkiewicz K Le Floc'h A Boutet M Vergnon I Schmitt A Mami-Chouaib F. CD103 or LFA-1 engagement at the immune synapse between cytotoxic T cells and tumor cells promotes maturation and regulates T-cell effector functions. Cancer Res (2013) 73:617–28. doi: 10.1158/0008-5472.CAN-12-2569 23188505

[24] Mackay LK Rahimpour A Ma JZ Collins N Stock AT Hafon ML. The developmental pathway for CD103(+)CD8+ tissue-resident memory T cells of skin. Nat Immunol (2013) 14:1294–301. doi: 10.1038/ni.2744 24162776

[25] Pan Y Tian T Park CO Lofftus SY Mei S Liu X. Survival of tissue-resident memory T cells requires exogenous lipid uptake and metabolism. Nature (2017) 543:252–6. doi: 10.1038/nature21379 PMC5509051 28219080

[26] Ray SJ Franki SN Pierce RH Dimitrova S Koteliansky V Sprague AG. The collagen binding α1β1 integrin VLA-1 regulates CD8 T cell-mediated immune protection against heterologous influenza infection. Immunity (2004) 20:167–79. doi: 10.1016/s1074-7613(04)00021-4 14975239

[27] Bromley SK Akbaba H Mani V Mora-Buch R Chasse AY Sama A. CD49a regulates cutaneous resident memory CD8(+) T cell persistence and response. Cell Rep (2020) 32:108085. doi: 10.1016/j.celrep.2020.108085 32877667 PMC7520726

[28] FitzPatrick MEB Provine NM Garner LC Powell K Amini A Irwin SL. Human intestinal tissue-resident memory T cells comprise transcriptionally and functionally distinct subsets. Cell Rep (2021) 34:108661. doi: 10.1016/j.celrep.2020.108661 33472060 PMC7816164

[29] Fung HY Teryek M Lemenze AD Bergsbaken T. CD103 fate mapping reveals that intestinal CD103- tissue-resident memory T cells are the primary responders to secondary infection. Sci Immunol (2022) 7:eabl9925. doi: 10.1126/sciimmunol.abl9925 36332012 PMC9901738

[30] von Hoesslin M Kuhlmann M de Almeida GP Kanev K Wurmser C Gerullis A-K. Secondary infections rejuvenate the intestinal CD103+ tissue-resident memory T cell pool. Sci Immunol (2022) 7:eabp9553. doi: 10.1126/sciimmunol.abp9553 36332011

[31] Fonseca R Burn TN Gandolfo LC Devi S Park SL Obers A. Runx3 drives a CD8(+) T cell tissue residency program that is absent in CD4(+) T cells. Nat Immunol (2022) 23:1236–45. doi: 10.1038/s41590-022-01273-4 PMC13045866 35882933

[32] Grueter B Petter M Egawa T Laule-Kilian K Aldrian CJ Wuerch A. Runx3 regulates integrin alpha E/CD103 and CD4 expression during development of CD4-/CD8+ T cells. J Immunol (Baltimore Md 1950) (2005) 175:1694–705. doi: 10.4049/jimmunol.175.3.1694 16034110

[33] Kurd NS He Z Louis TL Milner JJ Omilusik KD Jin W. Early precursors and molecular determinants of tissue-resident memory CD8(+) T lymphocytes revealed by single-cell RNA sequencing. Sci Immunol (2020) 5(47):eaaz6894. doi: 10.1126/sciimmunol.aaz6894 32414833 PMC7341730

[34] Wang D Diao H Getzler AJ Rogal W Frederick MA Milner J. The transcription factor Runx3 establishes chromatin accessibility of cis-regulatory landscapes that drive memory cytotoxic T lymphocyte formation. Immunity (2018) 48:659–74.e6. doi: 10.1016/j.immuni.2018.03.028 29669249 PMC6750808

[35] Milner JJ Toma C Yu B Zhang K Omilusik K Phan AT. Runx3 programs CD8(+) T cell residency in non-lymphoid tissues and tumours. Nature (2017) 552:253–7. doi: 10.1038/nature24993 PMC5747964 29211713

[36] Mackay LK Minnich M Kragten NA Liao Y Nota B Seillet C. Hobit and Blimp1 instruct a universal transcriptional program of tissue residency in lymphocytes. Sci (New York NY) (2016) 352:459–63. doi: 10.1126/science.aad2035 27102484

[37] Backer RA Helbig C Gentek R Kent A Laidlaw BJ Dominguez CX. A central role for notch in effector CD8(+) T cell differentiation. Nat Immunol (2014) 15:1143–51. doi: 10.1038/ni.3027 PMC4232996 25344724

[38] Wu J Madi A Mieg A Hotz-Wagenblatt A Weisshaar N Ma S. T Cell factor 1 suppresses CD103+ lung tissue-resident memory T cell development. Cell Rep (2020) 31:107484. doi: 10.1016/j.celrep.2020.03.048 32268106

[39] Hombrink P Helbig C Backer RA Piet B Oja AE Stark R. Programs for the persistence, vigilance and control of human CD8(+) lung-resident memory T cells. Nat Immunol (2016) 17:1467–78. doi: 10.1038/ni.3589 27776108

[40] Cruz-Guilloty F Pipkin ME Djuretic IM Levanon D Lotem J Lichtenheld MG. Runx3 and T-box proteins cooperate to establish the transcriptional program of effector CTLs. J Exp Med (2009) 206:51–9. doi: 10.1084/jem.20081242 PMC2626671 19139168

[41] Kragten NAM Behr FM Vieira Braga FA Remmerswaal EBM Wesselink TH Oja AE. Blimp-1 induces and hobit maintains the cytotoxic mediator granzyme b in CD8 T cells. Eur J Immunol (2018) 48:1644–62. doi: 10.1002/eji.201847771 30051906

[42] Lin YH Duong HG Limary AE Kim ES Hsu P Patel SA. Small intestine and colon tissue-resident memory CD8+ T cells exhibit molecular heterogeneity and differential dependence on eomes. Immunity (2023) 56(1):207–23.e8. doi: 10.1016/j.immuni.2022.12.007 PMC9904390 36580919

[43] Parga-Vidal L van Aalderen MC Stark R van Gisbergen K. Tissue-resident memory T cells in the urogenital tract. Nat Rev Nephrol (2022) 18:209–23. doi: 10.1038/s41581-021-00525-0 35079143

[44] Christo SN Evrard M Park SL Gandolfo LC Burn TN Fonseca R. Discrete tissue microenvironments instruct diversity in resident memory T cell function and plasticity. Nat Immunol (2021) 22:1140–51. doi: 10.1038/s41590-021-01004-1 34426691

[45] Parga-Vidal L Taggenbrock R Beumer-Chuwonpad A Aglmous H Kragten NAM Behr FM. Hobit and blimp-1 regulate TRM abundance after LCMV infection by suppressing tissue exit pathways of TRM precursors. Eur J Immunol (2022) 52:1095–111. doi: 10.1002/eji.202149665 PMC9545210 35389518

[46] Ryan GE Harris JE Richmond JM. Resident memory T cells in autoimmune skin diseases. Front Immunol (2021) 12:652191. doi: 10.3389/fimmu.2021.652191 34012438 PMC8128248

[47] Zundler S Becker E Spocinska M Slawik M Parga-Vidal L Stark R. Hobit- and blimp-1-driven CD4(+) tissue-resident memory T cells control chronic intestinal inflammation. Nat Immunol (2019) 20:288–300. doi: 10.1038/s41590-018-0298-5 30692620

[48] La Manna MP Di Liberto D Lo Pizzo M Mohammadnezhad L Shekarkar Azgomi M Salamone V. The abundance of tumor-infiltrating CD8(+) tissue resident memory T lymphocytes correlates with patient survival in glioblastoma. Biomedicines (2022) 10(10):2454. doi: 10.3390/biomedicines10102454 36289717 PMC9599482

[49] Lin R Zhang H Yuan Y He Q Zhou J Li S. Fatty acid oxidation controls CD8(+) tissue-resident memory T-cell survival in gastric adenocarcinoma. Cancer Immunol Res (2020) 8:479–92. doi: 10.1158/2326-6066.CIR-19-0702 32075801

[50] Byrne A Savas P Sant S Li R Virassamy B Luen SJ. Tissue-resident memory T cells in breast cancer control and immunotherapy responses. Nat Rev Clin Oncol (2020) 17:341–8. doi: 10.1038/s41571-020-0333-y 32112054

[51] Amsen D van Gisbergen KPJM Hombrink P van Lier RAW. Tissue-resident memory T cells at the center of immunity to solid tumors. Nat Immunol (2018) 19:538–46. doi: 10.1038/s41590-018-0114-2 29777219

[52] Okla K Farber DL Zou W. Tissue-resident memory T cells in tumor immunity and immunotherapy. J Exp Med (2021) 218(4):e20201605. doi: 10.1084/jem.20201605 33755718 PMC7992502

[53] Mami-Chouaib F Blanc C Corgnac S Hans S Malenica I Granier C. Resident memory T cells, critical components in tumor immunology. J Immunother Cancer (2018) 6:87. doi: 10.1186/s40425-018-0399-6 30180905 PMC6122734

[54] Mattei F Andreone S Marone G Gambardella AR Loffredo S Varricchi G. Eosinophils in the tumor microenvironment. Adv Exp Med Biol (2020) 1273:1–28. doi: 10.1007/978-3-030-49270-0_1 33119873

[55] Edwards J Wilmott JS Madore J Gide TN Quek C Tasker A. CD103(+) tumor-resident CD8(+) T cells are associated with improved survival in immunotherapy-naïve melanoma patients and expand significantly during anti-PD-1 treatment. Clin Cancer Res (2018) 24:3036–45. doi: 10.1158/1078-0432.Ccr-17-2257 29599411

[56] Murray T Fuertes Marraco SA Baumgaertner P Bordry N Cagnon L Donda A. Very late antigen-1 marks functional tumor-resident CD8 T cells and correlates with survival of melanoma patients. Front Immunol (2016) 7:573. doi: 10.3389/fimmu.2016.00573 28018343 PMC5150229

[57] Shen Y Li XL Li YX Shan ZG Zhao YL Cheng P. Distribution, phenotype, functional and clinical relevance of CD8(+)CD103(+) tissue-resident memory T cells in human gastric cancer. Cancer Immunol Immunother (2022) 71:1645–54. doi: 10.1007/s00262-021-03105-0 PMC10992218 34767045

[58] Li R Liu H Cao Y Wang J Chen Y Qi Y. Identification and validation of an immunogenic subtype of gastric cancer with abundant intratumoural CD103(+)CD8(+) T cells conferring favourable prognosis. Br J Cancer (2020) 122:1525–34. doi: 10.1038/s41416-020-0813-y PMC7217759 32205862

[59] Mori T Tanaka H Deguchi S Miki Y Yoshii M Tamura T. CD103(+) T cells may be a useful biomarker in borrmann type 4 gastric cancer. Cancer Diagn Progn (2022) 2:384–90. doi: 10.21873/cdp.10121 PMC9066542 35530656

[60] Webb JR Milne K Watson P Deleeuw RJ Nelson BH. Tumor-infiltrating lymphocytes expressing the tissue resident memory marker CD103 are associated with increased survival in high-grade serous ovarian cancer. Clin Cancer Res (2014) 20:434–44. doi: 10.1158/1078-0432.Ccr-13-1877 24190978

[61] Ganesan AP Clarke J Wood O Garrido-Martin EM Chee SJ Mellows T. Tissue-resident memory features are linked to the magnitude of cytotoxic T cell responses in human lung cancer. Nat Immunol (2017) 18:940–50. doi: 10.1038/ni.3775 PMC6036910 28628092

[62] Nizard M Roussel H Diniz MO Karaki S Tran T Voron T. Induction of resident memory T cells enhances the efficacy of cancer vaccine. Nat Commun (2017) 8:15221. doi: 10.1038/ncomms15221 28537262 PMC5458068

[63] Cheng Y Gunasegaran B Singh HD Dutertre CA Loh CY Lim JQ. Non-terminally exhausted tumor-resident memory HBV-specific T cell responses correlate with relapse-free survival in hepatocellular carcinoma. Immunity (2021) 54:1825–40.e7. doi: 10.1016/j.immuni.2021.06.013 34270940

[64] Barsch M Salié H Schlaak AE Zhang Z Hess M Mayer LS. T-Cell exhaustion and residency dynamics inform clinical outcomes in hepatocellular carcinoma. J Hepatol (2022) 77:397–409. doi: 10.1016/j.jhep.2022.02.032 35367533

[65] Yang R Cheng S Luo N Gao R Yu K Kang B. Distinct epigenetic features of tumor-reactive CD8+ T cells in colorectal cancer patients revealed by genome-wide DNA methylation analysis. Genome Biol (2019) 21:2. doi: 10.1186/s13059-019-1921-y 31892342 PMC6937914

[66] Luo Y Zong Y Hua H Gong M Peng Q Li C. Immune-infiltrating signature-based classification reveals CD103(+)CD39(+) T cells associate with colorectal cancer prognosis and response to immunotherapy. Front Immunol (2022) 13:1011590. doi: 10.3389/fimmu.2022.1011590 36311750 PMC9596778

[67] Malik BT Byrne KT Vella JL Zhang P Shabaneh TB Steinberg SM. Resident memory T cells in the skin mediate durable immunity to melanoma. Sci Immunol (2017) 2(10):eaam6346. doi: 10.1126/sciimmunol.aam6346 28738020 PMC5525335

[68] Enamorado M Khouili SC Iborra S Sancho D. Genealogy, dendritic cell priming, and differentiation of tissue-resident memory CD8(+) T cells. Front Immunol (2018) 9:1751. doi: 10.3389/fimmu.2018.01751 30108585 PMC6079237

[69] Vella JL Molodtsov A Angeles CV Branchini BR Turk MJ Huang YH. Dendritic cells maintain anti-tumor immunity by positioning CD8 skin-resident memory T cells. Life Sci alliance (2021) 4(10):e202101056. doi: 10.26508/lsa.202101056 34362825 PMC8356251

[70] Li X Zhai J Shen Y Zhang T Wang Y He Y. Tumor-derived IL-8 facilitates lymph node metastasis of gastric cancer *via* PD-1 up-regulation in CD8(+) T cells. Cancer Immunol Immunother (2022) 71:3057–70. doi: 10.1007/s00262-022-03223-3 PMC9588474 35633411

[71] Li Y Hu X Lin R Zhou G Zhao L Zhao D. Single-cell landscape reveals active cell subtypes and their interaction in the tumor microenvironment of gastric cancer. Theranostics (2022) 12:3818–33. doi: 10.7150/thno.71833 PMC9131288 35664061

[72] Farhood B Najafi M Mortezaee K. CD8(+) cytotoxic T lymphocytes in cancer immunotherapy: A review. J Cell Physiol (2019) 234:8509–21. doi: 10.1002/jcp.27782 30520029

[73] Beltra JC Manne S Abdel-Hakeem MS Kurachi M Giles JR Chen Z. Developmental relationships of four exhausted CD8(+) T cell subsets reveals underlying transcriptional and epigenetic landscape control mechanisms. Immunity (2020) 52:825–41 e8. doi: 10.1016/j.immuni.2020.04.014 32396847 PMC8360766

[74] Wherry EJ Ha SJ Kaech SM Haining WN Sarkar S Kalia V. Molecular signature of CD8+ T cell exhaustion during chronic viral infection. Immunity (2007) 27:670–84. doi: 10.1016/j.immuni.2007.09.006 17950003

[75] Wakim LM Woodward-Davis A Liu R Hu Y Villadangos J Smyth G. The molecular signature of tissue resident memory CD8 T cells isolated from the brain. J Immunol (2012) 189:3462–71. doi: 10.4049/jimmunol.1201305 PMC3884813 22922816

[76] Zheng L Qin S Si W Wang A Xing B Gao R. Pan-cancer single-cell landscape of tumor-infiltrating T cells. Science (2021) 374:abe6474. doi: 10.1126/science.abe6474 34914499

[77] Kim BS Kuen DS Koh CH Kim HD Chang SH Kim S. Type 17 immunity promotes the exhaustion of CD8(+) T cells in cancer. J immunother Cancer (2021) 9(6):e002603. doi: 10.1136/jitc-2021-002603 34083422 PMC8183213

[78] Sun K Xu R Ma F Yang N Li Y Sun X. scRNA-seq of gastric tumor shows complex intercellular interaction with an alternative T cell exhaustion trajectory. Nat Commun (2022) 13:4943. doi: 10.1038/s41467-022-32627-z 35999201 PMC9399107

[79] Guo X Zhang Y Zheng L Zheng C Song J Zhang Q. Global characterization of T cells in non-small-cell lung cancer by single-cell sequencing. Nat Med (2018) 24:978–85. doi: 10.1038/s41591-018-0045-3 29942094

[80] Milner JJ Toma C He Z Kurd NS Nguyen QP McDonald B. Heterogenous populations of tissue-resident CD8(+) T cells are generated in response to infection and malignancy. Immunity (2020) 52:808–24.e7. doi: 10.1016/j.immuni.2020.04.007 32433949 PMC7784612

[81] Clarke J Panwar B Madrigal A Singh D Gujar R Wood O. Single-cell transcriptomic analysis of tissue-resident memory T cells in human lung cancer. J Exp Med (2019) 216:2128–49. doi: 10.1084/jem.20190249 PMC6719422 31227543

[82] Anadon CM Yu X Hanggi K Biswas S Chaurio RA Martin A. Ovarian cancer immunogenicity is governed by a narrow subset of progenitor tissue-resident memory T cells. Cancer Cell (2022) 40:545–57 e13. doi: 10.1016/j.ccell.2022.03.008 35427494 PMC9096229

[83] Booth JS Toapanta FR Salerno-Goncalves R Patil S Kader HA Safta AM. Characterization and functional properties of gastric tissue-resident memory T cells from children, adults, and the elderly. Front Immunol (2014) 5:294. doi: 10.3389/fimmu.2014.00294 24995010 PMC4062881

[84] Li R Liu H Cao Y Wang J Chen Y Qi Y. Identification and validation of an immunogenic subtype of gastric cancer with abundant intratumoural CD103CD8 T cells conferring favourable prognosis. Br J Cancer (2020) 122:1525–34. doi: 10.1038/s41416-020-0813-y PMC7217759 32205862

[85] Lin R Zhang H Yuan Y He Q Zhou J Li S. Fatty acid oxidation controls CD8 tissue-resident memory T-cell survival in gastric adenocarcinoma. Cancer Immunol Res (2020) 8:479–92. doi: 10.1158/2326-6066.CIR-19-0702 32075801

[86] Mori T Tanaka H Suzuki S Deguchi S Yamakoshi Y Yoshii M. Tertiary lymphoid structures show infiltration of effective tumor-resident T cells in gastric cancer. Cancer Sci (2021) 112:1746–57. doi: 10.1111/cas.14888 PMC8088970 33735485

[87] Pan J Fan Z Wang Z Dai Q Xiang Z Yuan F. CD36 mediates palmitate acid-induced metastasis of gastric cancer *via* AKT/GSK-3beta/beta-catenin pathway. J Exp Clin Cancer Res (2019) 38:52. doi: 10.1186/s13046-019-1049-7 30717785 PMC6360779

[88] Aoki T Kinoshita J Munesue S Hamabe-Horiike T Yamaguchi T Nakamura Y. Hypoxia-induced CD36 expression in gastric cancer cells promotes peritoneal metastasis via fatty acid uptake. Ann Surg Oncol (2022) 10.1245/s10434-022-12465-5. doi: 10.1245/s10434-022-12465-5 36042102 PMC10085939

[89] Dieu-Nosjean MC Giraldo NA Kaplon H Germain C Fridman WH Sautès-Fridman C. Tertiary lymphoid structures, drivers of the anti-tumor responses in human cancers. Immunol Rev (2016) 271(1):260–75. doi: 10.1111/imr.12405 27088920

[90] Trüb M Zippelius A. Tertiary lymphoid structures as a predictive biomarker of response to cancer immunotherapies. Front Immunol (2021) 12:674565. doi: 10.3389/fimmu.2021.674565 34054861 PMC8149953

[91] Mori T Tanaka H Deguchi S Yamakoshi Y Miki Y Yoshii M. Clinical efficacy of nivolumab is associated with tertiary lymphoid structures in surgically resected primary tumors of recurrent gastric cancer. PloS One (2022) 17:e0262455. doi: 10.1371/journal.pone.0262455 34995329 PMC8741034

[92] Correa P. Gastric cancer: Overview. Gastroenterol Clin North Am (2013) 42:211–7. doi: 10.1016/j.gtc.2013.01.002 PMC3995345 23639637

[93] Machlowska J Baj J Sitarz M Maciejewski R Sitarz R. Gastric cancer: Epidemiology, risk factors, classification, genomic characteristics and treatment strategies. Int J Mol Sci (2020) 21(11):4012. doi: 10.3390/ijms21114012 32512697 PMC7312039

[94] Yang J Liu Z Zeng B Hu G Gan R. Epstein-Barr Virus-associated gastric cancer: A distinct subtype. Cancer Lett (2020) 495:191–9. doi: 10.1016/j.canlet.2020.09.019 32979463

[95] Deng R Zheng H Cai H Li M Shi Y Ding S. Effects of helicobacter pylori on tumor microenvironment and immunotherapy responses. Front Immunol (2022) 13:923477. doi: 10.3389/fimmu.2022.923477 35967444 PMC9371381

[96] Wu J Zhu X Guo X Yang Z Cai Q Gu D. Helicobacter urease suppresses cytotoxic CD8+ T-cell responses through activating Myh9-dependent induction of PD-L1. Int Immunol (2021) 33:491–504. doi: 10.1093/intimm/dxab044 34297096

[97] Xu N Ruan G Liu W Hu C Huang A Zeng Z. Vaccine-induced gastric CD4(+) tissue-resident memory T cells proliferate *in situ* to amplify immune response against helicobacter pylori insult. Helicobacter (2019) 24:e12652. doi: 10.1111/hel.12652 31414552

[98] Koch MR Gong R Friedrich V Engelsberger V Kretschmer L Wanisch A. CagA-specific gastric CD8+ tissue-resident T cells control helicobacter pylori during the early infection phase. Gastroenterology (2022). doi: 10.1053/j.gastro.2022.12.016 36587707

[99] Xing X Guo J Ding G Li B Dong B Feng Q. Analysis of PD1, PDL1, PDL2 expression and T cells infiltration in 1014 gastric cancer patients. Oncoimmunology (2018) 7:e1356144. doi: 10.1080/2162402X.2017.1356144 29399387 PMC5790386

[100] Sasaki S Nishikawa J Sakai K Iizasa H Yoshiyama H Yanagihara M. EBV-associated gastric cancer evades T-cell immunity by PD-1/PD-L1 interactions. Gastric Cancer (2019) 22:486–96. doi: 10.1007/s10120-018-0880-4 30264329

[101] Peng R Liu S You W Huang Y Hu C Gao Y. Gastric microbiome alterations are associated with decreased CD8+ tissue-resident memory T cells in the tumor microenvironment of gastric cancer. Cancer Immunol Res (2022) 10:1224–40. doi: 10.1158/2326-6066.Cir-22-0107 35881964

[102] Yang L He YT Dong S Wei XW Chen ZH Zhang B. Single-cell transcriptome analysis revealed a suppressive tumor immune microenvironment in EGFR mutant lung adenocarcinoma. J immunother Cancer (2022) 10(2):e003534. doi: 10.1136/jitc-2021-003534 35140113 PMC8830346

[103] Lutter L Roosenboom B Brand EC Ter Linde JJ Oldenburg B van Lochem EG. Homeostatic function and inflammatory activation of ileal CD8(+) tissue-resident T cells is dependent on mucosal location. Cell Mol Gastroenterol Hepatol (2021) 12:1567–81. doi: 10.1016/j.jcmgh.2021.06.022 PMC8551698 34224909

[104] Wang C Kong L Kim S Lee S Oh S Jo S. The role of IL-7 and IL-7R in cancer pathophysiology and immunotherapy. Int J Mol Sci (2022) 23(18):10412. doi: 10.3390/ijms231810412 36142322 PMC9499417

[105] de Andrea CE Schalper KA Sanmamed MF Melero I. Immunodivergence in metastatic colorectal cancer. Cancer Cell (2018) 34:876–8. doi: 10.1016/j.ccell.2018.11.012 PMC7385539 30537510

[106] Zhang N Bevan MJ. Transforming growth factor-beta signaling controls the formation and maintenance of gut-resident memory T cells by regulating migration and retention. Immunity (2013) 39:687–96. doi: 10.1016/j.immuni.2013.08.019 PMC3805703 24076049

[107] Lange J Rivera-Ballesteros O Buggert M. Human mucosal tissue-resident memory T cells in health and disease. Mucosal Immunol (2022) 15:389–97. doi: 10.1038/s41385-021-00467-7 PMC8571012 34743182

[108] Chen B Mu C Zhang Z He X Liu X. The love-hate relationship between TGF-beta signaling and the immune system during development and tumorigenesis. Front Immunol (2022) 13:891268. doi: 10.3389/fimmu.2022.891268 35720407 PMC9204485

[109] Jiang W Wang X Zeng B Liu L Tardivel A Wei H. Recognition of gut microbiota by NOD2 is essential for the homeostasis of intestinal intraepithelial lymphocytes. J Exp Med (2013) 210:2465–76. doi: 10.1084/jem.20122490 PMC3804938 24062413

[110] Patidar M Yadav N Dalai SK. Interleukin 15: A key cytokine for immunotherapy. Cytokine Growth factor Rev (2016) 31:49–59. doi: 10.1016/j.cytogfr.2016.06.001 27325459

[111] Cui F Qu D Sun R Zhang M Nan K. NK cell-produced IFN-γ regulates cell growth and apoptosis of colorectal cancer by regulating IL-15. Exp Ther Med (2020) 19:1400–6. doi: 10.3892/etm.2019.8343 PMC6966233 32010315

[112] Bahri R Pateras IS D'Orlando O Goyeneche-Patino DA Campbell M Polansky JK. IL-15 suppresses colitis-associated colon carcinogenesis by inducing antitumor immunity. Oncoimmunology (2015) 4:e1002721. doi: 10.1080/2162402x.2014.1002721 26405589 PMC4570106

[113] Desbois M Le Vu P Coutzac C Marcheteau E Beal C Terme M. IL-15 trans-signaling with the superagonist RLI promotes Effector/Memory CD8+ T cell responses and enhances antitumor activity of PD-1 antagonists. J Immunol (Baltimore Md 1950) (2016) 197:168–78. doi: 10.4049/jimmunol.1600019 27217584

[114] Bui QL Mas L Hollebecque A Tougeron D de la Fouchardiere C Pudlarz T. Treatments after immune checkpoint inhibitors in patients with dMMR/MSI metastatic colorectal cancer. Cancers (Basel) (2022) 14(2):406. doi: 10.3390/cancers14020406 35053568 PMC8774125

[115] Randrian V Evrard C Tougeron D. Microsatellite instability in colorectal cancers: Carcinogenesis, neo-antigens, immuno-resistance and emerging therapies. Cancers (Basel) (2021) 13(12):3063. doi: 10.3390/cancers13123063 34205397 PMC8235567

[116] De' Angelis GL Bottarelli L Azzoni C De' Angelis N Leandro G Di Mario F. Microsatellite instability in colorectal cancer. Acta BioMed (2018) 89:97–101. doi: 10.23750/abm.v89i9-S.7960 30561401 PMC6502181

[117] Guastadisegni C Colafranceschi M Ottini L Dogliotti E. Microsatellite instability as a marker of prognosis and response to therapy: A meta-analysis of colorectal cancer survival data. Eur J Cancer (Oxford Engl 1990) (2010) 46:2788–98. doi: 10.1016/j.ejca.2010.05.009 20627535

[118] de Vries NL van Unen V Ijsselsteijn ME Abdelaal T van der Breggen R Farina Sarasqueta A. High-dimensional cytometric analysis of colorectal cancer reveals novel mediators of antitumour immunity. Gut (2020) 69:691–703. doi: 10.1136/gutjnl-2019-318672 31270164 PMC7063399

[119] Duhen T Duhen R Montler R Moses J Moudgil T de Miranda NF. Co-Expression of CD39 and CD103 identifies tumor-reactive CD8 T cells in human solid tumors. Nat Commun (2018) 9:2724. doi: 10.1038/s41467-018-05072-0 30006565 PMC6045647

[120] Sung H Ferlay J Siegel RL Laversanne M Soerjomataram I Jemal A. Global cancer statistics 2020: GLOBOCAN estimates of incidence and mortality worldwide for 36 cancers in 185 countries. CA Cancer J Clin (2021) 71:209–49. doi: 10.3322/caac.21660 33538338

[121] Baidoun F Elshiwy K Elkeraie Y Merjaneh Z Khoudari G Sarmini MT. Colorectal cancer epidemiology: Recent trends and impact on outcomes. Curr Drug Targets (2021) 22:998–1009. doi: 10.2174/1389450121999201117115717 33208072

[122] Tran E Robbins PF Lu YC Prickett TD Gartner JJ Jia L. T-Cell transfer therapy targeting mutant KRAS in cancer. N Engl J Med (2016) 375:2255–62. doi: 10.1056/NEJMoa1609279 PMC5178827 27959684

[123] Edwards J Wilmott JS Madore J Gide TN Quek C Tasker A. CD103+ tumor-resident CD8+ T cells are associated with improved survival in immunotherapy-naïve melanoma patients and expand significantly during anti–PD-1 treatment. Clin Cancer Res (2018) 24:3036–45. doi: 10.1158/1078-0432.Ccr-17-2257 29599411

[124] Luoma AM Suo S Wang Y Gunasti L Porter CBM Nabilsi N. Tissue-resident memory and circulating T cells are early responders to pre-surgical cancer immunotherapy. Cell (2022) 185:2918–35.e29. doi: 10.1016/j.cell.2022.06.018 35803260 PMC9508682

[125] Enamorado M Iborra S Priego E Cueto FJ Quintana JA Martínez-Cano S. Enhanced anti-tumour immunity requires the interplay between resident and circulating memory CD8(+) T cells. Nat Commun (2017) 8:16073. doi: 10.1038/ncomms16073 28714465 PMC5520051

[126] Lauret Marie Joseph E Kirilovsky A Lecoester B El Sissy C Boullerot L Rangan L. Chemoradiation triggers antitumor Th1 and tissue resident memory-polarized immune responses to improve immune checkpoint inhibitors therapy. J immunother Cancer (2021) 9(7):e002256. doi: 10.1136/jitc-2020-002256 34230108 PMC8261891

[127] Janjigian YY Shitara K Moehler M Garrido M Salman P Shen L. First-line nivolumab plus chemotherapy versus chemotherapy alone for advanced gastric, gastro-oesophageal junction, and oesophageal adenocarcinoma (CheckMate 649): a randomised, open-label, phase 3 trial. Lancet (London England) (2021) 398:27–40. doi: 10.1016/s0140-6736(21)00797-2 34102137 PMC8436782

[128] Wu TC Xu K Banchereau R Marches F Yu CI Martinek J. Reprogramming tumor-infiltrating dendritic cells for CD103+ CD8+ mucosal T-cell differentiation and breast cancer rejection. Cancer Immunol Res (2014) 2:487–500. doi: 10.1158/2326-6066.Cir-13-0217 24795361 PMC4014008

[129] Kobayashi M Sakabe T Chiba A Nakajima A Okamoto M Shimodaira S. Therapeutic effect of intratumoral injections of dendritic cells for locally recurrent gastric cancer: a case report. World J Surg Oncol (2014) 12:390. doi: 10.1186/1477-7819-12-390 25526950 PMC4320508

[130] Rosato PC Wijeyesinghe S Stolley JM Nelson CE Davis RL Manlove LS. Virus-specific memory T cells populate tumors and can be repurposed for tumor immunotherapy. Nat Commun (2019) 10:567. doi: 10.1038/s41467-019-08534-1 30718505 PMC6362136

[131] Sandoval F Terme M Nizard M Badoual C Bureau MF Freyburger L. Mucosal imprinting of vaccine-induced CD8^+^ T cells is crucial to inhibit the growth of mucosal tumors. Sci Trans Med (2013) 5:172ra20. doi: 10.1126/scitranslmed.3004888 PMC4086646 23408053

[132] Cafri G Gartner JJ Zaks T Hopson K Levin N Paria BC. mRNA vaccine-induced neoantigen-specific T cell immunity in patients with gastrointestinal cancer. J Clin Invest (2020) 130:5976–88. doi: 10.1172/jci134915 PMC7598064 33016924

[133] Besser MJ Shapira-Frommer R Treves AJ Zippel D Itzhaki O Hershkovitz L. Clinical responses in a phase II study using adoptive transfer of short-term cultured tumor infiltration lymphocytes in metastatic melanoma patients. Clin Cancer Res (2010) 16:2646–55. doi: 10.1158/1078-0432.Ccr-10-0041 20406835

[134] Zacharakis N Huq LM Seitter SJ Kim SP Gartner JJ Sindiri S. Breast cancers are immunogenic: Immunologic analyses and a phase II pilot clinical trial using mutation-reactive autologous lymphocytes. J Clin Oncol (2022) 40:1741–54. doi: 10.1200/jco.21.02170 PMC9148699 35104158

[135] Morgan RA Yang JC Kitano M Dudley ME Laurencot CM Rosenberg SA. Case report of a serious adverse event following the administration of T cells transduced with a chimeric antigen receptor recognizing ERBB2. Mol Ther (2010) 18:843–51. doi: 10.1038/mt.2010.24 PMC2862534 20179677

[136] Zheng C Fass JN Shih YP Gunderson AJ Sanjuan Silva N Huang H. Transcriptomic profiles of neoantigen-reactive T cells in human gastrointestinal cancers. Cancer Cell (2022) 40:410–23.e7. doi: 10.1016/j.ccell.2022.03.005 35413272

[137] He J Xiong X Yang H Li D Liu X Li S. Defined tumor antigen-specific T cells potentiate personalized TCR-T cell therapy and prediction of immunotherapy response. Cell Res (2022) 32:530–42. doi: 10.1038/s41422-022-00627-9 PMC9160085 35165422

[138] Molodtsov AK Khatwani N Vella JL Lewis KA Zhao Y Han J. Resident memory CD8(+) T cells in regional lymph nodes mediate immunity to metastatic melanoma. Immunity (2021) 54:2117–32.e7. doi: 10.1016/j.immuni.2021.08.019 34525340 PMC9015193

